# Early detection of oral cancer: PAP and AgNOR staining in brush biopsies

**DOI:** 10.4103/0973-029X.72501

**Published:** 2010

**Authors:** Dinesh V Rajput, Jagdish V Tupkari

**Affiliations:** *Department of Oral Pathology and Microbiology, YCMM and RDF’s Dental College and Hospital, Wadgaon Gupta MIDC, Ahmednagar - 414001, India*; 1 *Department of Oral Pathology and Microbiology, Government Dental College and Hospital, Mumbai - 400001, Maharashtra, India*

**Keywords:** AgNOR analysis, brush biopsy, cancer screening, diagnostic accuracy, early cancer detection, nucleolar organizer regions, oral cancer

## Abstract

**Aim:**

The aim of this study was to determine the diagnostic accuracy of routine Papanicolaou stain (PAP) and Silver stained Nucleolar Organizer Regions (AgNOR) staining in brush biopsies taken from suspected oral lesions for early detection of oral cancer.

**Materials and Methods:**

Brush biopsies were collected from macroscopically suspicious lesions of the oral cavity of 34 patients and 10 normal-aged and sex-matched controls. The numbers of AgNORs were counted in 100 squamous epithelial cell nuclei per slide after silver staining of the smears (Ploton’s one-step method).

**Results:**

Sensitivity and specificity of PAP analysis in the oral smears for detection of oral cancer and normal cells was 91.176% and 100%. The positive and negative prediction values were 100% and 76.92%, respectively. Sensitivity and specificity of AgNOR analysis in the oral smears for detection of oral cancer and normal cells was 100%. The positive and negative prediction values were 100% each.

**Conclusion:**

Based on the above facts, we conclude that brush biopsy in conjunction with AgNOR staining is an easily practicable, non-invasive, safe and accurate screening method for the detection of macroscopically suspicious oral cancerous lesions. Because of its simple technique and high reliability for cellular proliferation, AgNOR staining in brush smears can be used as an adjunct to other routine cytological diagnoses for the early detection of oral cancer. However, further investigations with more number of study samples will be needed to establish this correlation beyond doubt.

## INTRODUCTION

Oral squamous cell carcinomas (OSCCs) currently hold the sixth position in the worldwide cancer statistics,[1] with a dismal 5-year survival rate, except when diagnosed in the early stages.[2] Hence, there is a need to promote early diagnosis of oral cancers.[3] But, the only established method for their diagnosis is biopsy, which is carried out only when the lesions become symptomatic, i.e. in the late/advanced stages.[4]

Exfoliative cytology is an easy, non-invasive procedure and hence could be carried out even on slightest suspicion regarding the nature of the given lesion.[2] Although the reliability of oral exfoliative cytology has been questioned by many studies,[5] interest in this technique has been renewed due to the advent of newer modifications, like cytobrush and image analysis systems.[6] But, image analysis systems are not available in all institutes because of their high cost and the need of well-trained labor.[3] Therefore, our aim was to make the diagnostic procedures simpler and inexpensive and, at the same time, to increase the sensitivity and specificity of the routine exfoliative cytology.

In the last few years, AgNOR analysis is being frequently used to determine the prognosis of many malignant lesions.[7] NORs can be identified indirectly by means of argyrophilia of their associated proteins (AgNORs) as nuclear dark dots. Many recent reports have suggested that the number of AgNORs per nucleus is related to cellular proliferation and differentiation. This finding could be useful in differentiating between normal, benign and malignant lesions.[8] In addition, because this technique can be carried out with basic laboratory facilities and a light microscope, it will be helpful in increasing the sensitivity and specificity of exfoliative cytology.[9]

The purpose of this study was to determine the diagnostic accuracy of routinely performed PAP staining as compared with AgNOR staining in brush biopsies of suspected oral lesions.

## MATERIALS AND METHODS

### Subject population

This study was carried out in the Department of Oral Pathology and Microbiology, Government Dental College and Hospital, Aurangabad. The study population consisted of 44 subjects (including control), from which 88 smears (two smears per subject) were obtained. After thorough evaluation, 10 subjects for the control group were selected from age- and sex-matched subjects with prior consent and these subjects comprised the group I category of this study.

The 34 subjects having clinically diagnosed or suspicious of cancerous lesions (excluding recurrent lesions or those who had taken some sort of treatment) were grouped separately and comprised the group II category of this study. The brush biopsies were obtained and diagnosed before scalpel biopsies clarified the nature of the oral lesions histologically. The quantification of AgNOR counts was performed blindly without the knowledge of the cytological or histopathological report.

### Clinical procedure

After thorough clinical examination and consent, the subjects were subjected to 5-min gargling and the lesional areas were wiped off of excessive saliva and surface debris using a moistened gauze piece. Lesional areas with erythematous patches were usually preferred as collection sites. In case of highly keratotic or exophytic lesions, fissured or ulcerative areas were preferred for collecting the cells.

Two cytologic smears were obtained from the pathologic area in question using a cytobrush plus GT (cytobrush GT plus, Med-Scand Medical, Malmo, Sweden). The head of the cytobrush cell collector was moistened with water and was then firmly held against the mucosa of the lesional area. Then, gentle pressure was applied to the brush until the bristles curled or tiny bleeding spots were evident. In this position, the brush was rolled over the lesional site and was rotated for 10 full turns. The cytobrush cell collector was then rolled on glass slides by applying a continuous motion from one end of the slide to the other.

### Staining and mode of interpretation

The spray-fixed smears were stained by a commercially available RAPID-PAP Papanicolaou stain kit (Biolab Diagnostics, Boisar, Maharashtra, India).

**Figure 1:**
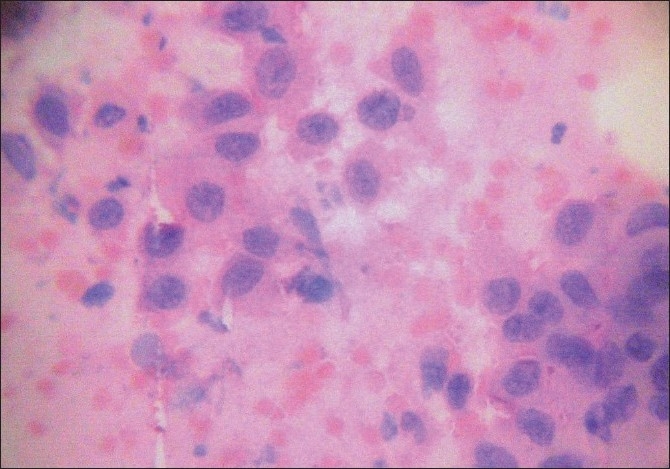
Class V cytology showing dysplastic epithelial cells (PAP stain, ×400 magnification)

Evaluation of Papanicolaou the-stained smears was carried out according to the standardized procedure at a magnification of ×450. The cells suspected to be abnormal were evaluated at higher magnifications and the location of abnormal cells was marked on the cover slip by an ink dot [Figure 1].

### AgNOR staining

AgNOR staining was performed according to the one-step method of Ploton *et al*.[10] with slight modifications suggested by Linder.[11] Further, some modifications were made in the staining procedure so as to suit the laboratory conditions.

### Procedure

The alcohol-fixed smears were immersed in 95% absolute ethanol followed by progressive rehydration and washing in distilled water. The AgNOR staining was carried out using a solution containing one part of 2% gelatine in 1% aqueous formic acid and two parts of 50% silver nitrate. The smears were flooded with silver-colloidal mixture and were kept under safelight conditions for 55 min at room temperature. After staining, the smears were placed in a dark container and washed in three changes of deionised water, followed by immersion in 5% sodium thiosulfate solution for 5 min. After thoroughly washing the smears in running tap water for 5 min, they were immersed in hypo eliminator solution for 5 min. After this step, the smears were washed in several changes of distilled water. This was followed by sequential dehydration in graded alcohols, cleared in xylene and mounted in synthetic medium (DPX).

### Results

**Figure 2:**
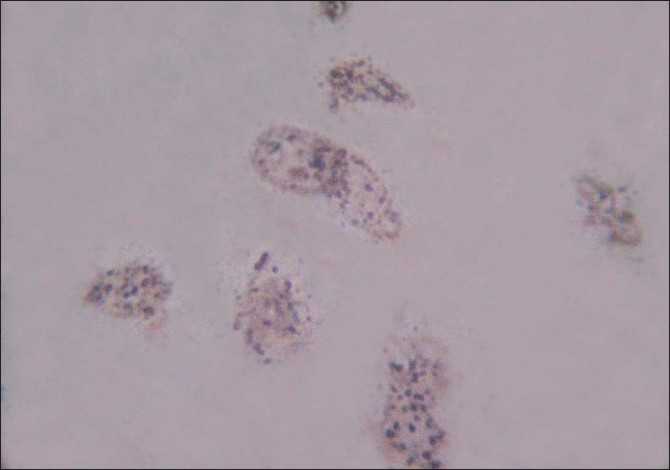
AgNORs in dysplastic epithelial cells of oral squamous cell carcinoma (AgNOR stain, ×1000 magnification)

Nucleolar organizer regions appeared as brown to black dots [Figure 2].

### Counting procedure

AgNOR counting was carried out according to Crocker’smethod.[12] All smears were examined under X1000 magnification in oil immersion using a Labomed Binocular microscope HL-AT1-B, Labo America, Inc, Auburn court, Fremont, CA, USA). In all the sections, the argyrophilic NORs were distinctly visible as dark brown to black “dots” or “blebs” of varying size in the brown-stained nucleus on a pale yellow background of the cells [Figure 2]. The overall slide background was clear. However, a small amount of extraneous silver deposits were present. To standardize the procedure of counting, the following steps were taken:

1. Firstly, all silver-stained structures were counted, both lying in groups (clusters) and as individual dots outside the clusters.
2. Secondly, the number of the clusters per nucleus was counted. The partly disaggregated dots associated with the clusters were considered as one structure.
3. Finally, just the numbers of individual dots (satellites) were counted.

The mean number of AgNORs per nucleus as clusters, as satellites, as clusters and satellites together and all AgNORs lying together in clusters and as satellites were calculated in each case.

## RESULTS

### PAP staining evaluation

Of technically sufficient brushings from the study sample, all 10 cases in the control group were classed as Class I, i.e. normal cytology. In the second group, a diagnosis of positive for malignancy was made in nine subjects [Figure 1] and 15 subjects were suggestive of the presence of malignancy. The diagnosis of intermediate cytology (Class III) was made in seven subjects, which is indicative of severe dysplasia or carcinoma *in situ*. The diagnosis of Class II cytology was made in three subjects.

Of the nine cases positive for malignancy, all were histologically proved to be OSCC. Of the 15 cases suggestive of malignancy, all were histologically proved to be OSCC. Of seven cases of intermediate cytology, six were histologically proven to be OSCC and one was diagnosed as verrucous carcinomas. Of the three cases of Class II cytology, all were histologically proven to be verrucous carcinoma.

Sensitivity of our PAP analysis in oral smears for the detection of oral cancer was 91.176%, while specificity for the detection of non-neoplastic cells was 100%. The positive and negative predictive values were 100% and 76.92%, respectively [Table 1].

**Table 1:** Sensitivity & andspecificity of PAP staining versus histology in OSCC and verrucous carcinoma

PAP grade	No. of cases	Histopathology			+ve PV 100%	-ve PV 76.92%
		OSCC		Verrucous carcinoma	
		+ve for tumor cells	-ve for tumor cells		
Class I	10	10	-	-	-	-
Class II	3	3	-	3	-	-
Class III	7	-	6	1	-	-
Class IV	15	-	15	-	-	-
Class V	9	-	9	-	-	-
Total	44	13	30	4	-	-

### AgNOR evaluation

In the healthy control group, the epithelial cells revealed 2.07–3.05 NORs per nucleus (mean, 2.568±0.3178). In the verrucous carcinoma group, the epithelial cells revealed 4–4.46 NORs per nucleus (mean, 4.223±0.1902). In the OSCC group, the number of NORs per epithelial cell ranged from 4.83 to 6.09 (mean, 5.384±0.3444) [Table 2] [Figure 2]. The cut-off value to differentiate between normal and malignant cells was four.

**Table 2:** mAgNOR counts in the study groups

Group	*n* = x	Count/nucleus		Mean count/nucleus		Pooled mean (±SD)
		Min.	Max.	Min.	Max.	
Control	10	1	6	2.07	3.05	2.568 (±0.3178)
Verrucous carcinoma	4	1	8	4	4.60	4.223 (±0.1902)
OSCC	30	1	11	4.83	6.09	5.384 (±0.3444)

In the healthy control group, more numbers of clusters were observed, ranging from 118 to 192 clusters per 100 cells, while the number of clusters in the verrucous carcinomas and OSCC ranged from 91 to 177 and 78 to 113, respectively. The number of satellites in healthy controls, verrucous carcinomas and OSCC were 49–158, 308–368 and 308–489 per 100 cells, respectively. Hence, in the control group, the number of NORs in the form of clusters was more than those in the form of satellites, while in the verrucous and OSCC groups, the number of satellites was significantly higher than that in the control group.

Histologically, in Group II, four subjects were proved to have verrucous carcinoma and 30 subjects had OSCC. The sensitivity of our AgNOR analysis in oral smears for the detection of oral cancer was 100% whereas the specificity for the detection of non-neoplastic cells was 100%. The positive and negative predictive values were 100% each [Table 3].

**Table 3:** Sensitivity and specificity of mAgNOR counts versus histopathology in the study groups

mAgNOR	No. of cases	Histology		Positive predictive value	Negative predictive value
		Negative for tumor cells	Positive for tumor cells		
<4	10	10	0	100%	100%
>4	34	0	34		

### Statistical analysis

Correlation between various cytological grades obtained by routine PAP method and histological grading was determined by a two-way variance ANOVA test. The *P*-value was more than 0.05. Hence, the correlation between cytological grades and histological grading was not significant. Correlation between mAgNOR counts and control, verrucous and OSCC groups determined by means of a one-way variance ANOVA test. The mAgNOR counts were found to increase in number from the control group to the OSCC group. The *P*-value was <0.05. Hence, the correlation was extremely significant [Table 4].

**Table 4:** Statistical correlation between AgNOR counts in the different groups

Groups	mAgNORs (±SD)	Degree of freedom	F-value	*P*-value	Statistical significance
Control	2.568 (±0.3178)	2	277	0.0001	Extremely significant
Verrucous carcinoma	4.223 (±0.1902)	-	-	-	-
OSCC	5.384 (±0.3444)	-	-	-	-

## DISCUSSION

Early detection of OSCCs not only increases the survival rate but also reduces the need for disfiguring treatments. Unfortunately, early detection of oral cancerous lesions has proved difficult because as many as 50% of the patients have regional or distant metastases at the time of diagnosis.[2,3]

The malignant transformation at the beginning of carcinogenesis affects only few cells long before small parts of tissue are involved.[3,4] Thus, cytologic examination should be a suitable method to elucidate the dignity of suspicious oral lesions earlier than histology, especially when used with sensitive markers like AgNORs.

Clinical examination and histopathological studies of biopsied material are the classical diagnostic methods used for the diagnosis of oral cancerous lesions. Biopsy is a “bloody” technique with surgical implications, technique limitations for some professionals and psychological implications for some patients. It also presents limitations when the lesions are large as, in these cases, it is important to select the more appropriate place where histological changes would be present. Even though biopsic study is fundamental, it is a diagnostic method with limited sensitivity, where one of the most important features is the subjectivity of the pathologists, which has been proved by few reports.[13–16] The diagnostic reliability of incisional biopsies has not been proved by any scientific study. Yet, despite this, it is accepted worldwide as a reliable way to obtain oral diagnosis, both in major textbooks.[17,18] and in national protocols.[4,19,20]

In contrast, exfoliative cytology is an easy, reliable technique that could be beneficial for the early diagnosis of oral cancers.[21] However, this technique is marred with a high false-negative rate (range, 0–31%).[22] One of the most common failures of exfoliative cytology in previous studies was the faulty techniques of smear collection, often yielding insufficient quantity of cells as required for microscopic examination.[14,23]

These flaws can very well be removed by using a more reliable sample collector like cytobrush. The superiority of the cytobrush with regards sample collection from a given lesion has been proved by many studies.[24,25] The cytobrush seems to have many advantages over traditional sample collectors with regard to cell yield and dispersion. By using a cytobrush, more number of cells are collected and can be more evenly spread over a slide thus allowing an easier interpretation.[25] In this study, all smears collected contained more than sufficient cells required for microscopic analysis.

Another common flaw of exfoliative cytology is subjectivity in interpretation of the given sample when stained by the routine PAP method. Cytological diagnoses of oral cancer cells are difficult and need much experience. Its application is thus limited by the requirement of highly specialized cytopathologists. However, recently, the introduction of molecular markers, image analysis systems and proliferation markers has helped to eliminate the above error. However, these techniques are rather expensive and time consuming and facilities required for them are not available in all institutions.[26]

Deregulated proliferation is considered to be a prime characteristic of malignancies. However, the estimation of proliferation in clinical material is still problematic. In contrast to several techniques that merely estimate static parameters of proliferation, such as the percentage of cycling or S-phase cells, AgNORs seem to reflect dynamic aspects of the cell cycle, i.e., the rapidity of cell duplication.[27] NORs are the morphologic sites around which the nucleolus develops at the end of mitosis. *In situ* hybridization techniques have shown that these regions represent the loops of DNA actively transcribing to rRNA and thus to ribosomes and, ultimately, to proteins. NORs can be identified by means of the argyrophilia of their associated proteins (NORAPs) as nuclear dark dots. Most recent reports have suggested that the number of AgNORs per nucleus is related to cellular proliferation and differentiation. These findings suggest that AgNORs can be used as an aid in diagnosing malignant lesions such as OSCC.[8,28] Hence, the aim of present study was to investigate whether the AgNOR method could play a role in the diagnosis of OSCC by studying exfoliated cells from suspected lesions using the cytobrush technique.

The method is applicable with simple light microscopes without additional and expensive technical options and hence could prove a cheap yet specific marker for proliferation when used in smears [Figure 2].[26,28] This property of AgNOR is very useful because the false-negative rate of cytologic examination alone could be as high as 31%.[22] Based on these facts, one aim of the present study was to compare the diagnostic accuracy of routine PAP staining and AgNOR staining in exfoliated cells of suspicious oral lesions. The advantage of using exfoliated cells for AgNOR counting is that the whole cell can be examined, reducing the possibility of underestimating the AgNOR counts per nucleus. The risk of obscuring some AgNORs by superimposition and coalescence is minimal.[8] Many papers have reported about the diagnostic values of AgNOR staining in OSCCs using biopsy specimens.[27,29–31] However, only few investigators have dealt with AgNOR on cytologic material (scrapings or brushings) of the oral cavity. Mao[8] reported the mean AgNOR counts per nucleus in exfoliated cells of the cancer group at 4.69±0.72 and 2.44±0.37 for normal mucosa. His AgNOR counts showed that the mean value for cancerous lesions were significantly higher than those of the normal mucosa (*P*<0.005). He found no overlap between the two groups. The results of the present study are basically in agreement with the data of Mao. In the control group, the mAgNOR count was 2.568 (±0.3178); in verrucous carcinoma, the mAgNOR count was 4.223 (±0.1902); and in the OSCC group, the mAgNOR count was 5.384 (0.3444). The mAgNOR counts were significantly different in all groups, the *P*-value being <0.005. The mAgNOR counts in the present study were slightly higher than those reported by Mao, which may be related to the advanced grades of lesions and/or due to racial variations.

Remmerbach *et al*.^26^ compared the mAgNOR and pAgNOR counts in control, benign inflammatory lesions, oral leukoplakia and OSCCs by using the exfoliated cells. The mAgNOR counts reported by these authors were 2.31 (±1.7), 3.39 (±0.4), 3.88 (±0.59) and 8.99 (±2.64), respectively. The mAgNOR counts of these authors were significantly greater than the mAgNOR counts obtained in the present study. This may be attributed to the staining time used in these studies. Remmerbach *et al*. stained the smears only for 20 min while in the present study, the staining time used was 55 min. Because of an increase in the staining time in the present study, the small NORs present in the given nucleus may have fused with each other due to continued deposition of silver for a longer time. As a result, the small-sized dot-like precipitations merge and the discrimination between small individual dots may not be possible.

In the present study, the diagnostic accuracy of routine PAP method and AgNOR staining were compared for the diagnosis of OSCCs. The routine PAP-stained smears were graded into five grades based on Papanicolaou’s criteria.[22] Thus, the sensitivity of PAP staining was 91.176% and the specificity was 100%. Based on these facts, the negative and positive predictive values were 76.92% and 100%, respectively.

The false-negative rate of PAP staining for diagnosing OSCCs in the present study was 23.08%. Folsom *et al*. reported the false-negative rate of exfoliative cytology to be ranging from 0% to 29% and, in his own study, the rate was 31%.[22] The false-negative rate in the present study was within the above range.

Scuibba[2] and Remmerbach *et al*.[26] have reported false-negative rates of 4%and 1.5%, respectively, which is significantly less than that in the present study. However, the above authors had used image analysis programs to analyze the brush smears. The use of such programs makes identification of abnormal cells easier, quicker and less tedious, which could be responsible for the lower false-negative rates.

Furthermore, epithelial dysplasias and borderline lesions represent morphological alterations that are suspicious for malignancy, but do not provide sufficient evidence for its definitive diagnosis.[3] This may be applicable to the present study because the subjects who were reported as false negative were histologically confirmed as having verrucous carcinoma. The grade of malignancy in this lesion may have been very low, which in turn had resulted in a false-negative diagnosis.

On the other hand, the latter situation offers the opportunity for adjuvant methods to identify the malignancy earlier than subjective interpretation of histological or cytological images. Here lies the importance of AgNOR analysis, which, due to its simplicity, has an edge over immunohistochemical methods for the similar purpose. When AgNOR staining was employed in the present study, the sensitivity and specificity of AgNOR staining was 100%. The negative and predictive values were also 100%. Here lies the advantage of AgNOR analysis because, unlike the PAP method, this analysis was able to not only diagnose all cases of verrucous carcinomas but also differentiate them from OSCCs. Hence, NOR analysis may be useful as a quantitative marker of incipient cellular alterations even before the histological hallmarks of changes can be detected.[30]

Based on the above facts, AgNOR analysis appears to be far superior to routine PAP staining when used for the detection of OSCCs in exfoliated cells.

## CONCLUSION

All smears contained more than sufficient number of cells required for microscopic analysis and from all cell layers. This proves the efficiency of the cytobrush cell collector. The mAgNOR per nucleus is a reliable marker of neoplastic squamous cells in oral smears. This method is able to increase the sensitivity for the detection of malignant and specificity for benign cells in oral smears and, thus, decreases the rate of cytologically false-negative or positive diagnoses. The AgNOR technique in exfoliative cytology can be used as an adjuvant diagnostic aid to routine cyto- and histopathology for differentiating between benign and malignant lesions of the oral cavity.

Although there seems to be a generally positive correlation between AgNOR counts and degree of malignancy, further investigations with more number of study samples will be needed to establish this correlation beyond doubt. However, their ease of demonstration and high specificity to cellular proliferation makes them the best available cytopathological marker in the arsenal of the oral pathologist.
